# When and where? Pathogenic *Escherichia coli* differentially sense host D-serine using a universal transporter system to monitor their environment

**DOI:** 10.15698/mic2016.04.494

**Published:** 2016-03-31

**Authors:** James P. R. Connolly, Andrew J. Roe

**Affiliations:** 1 Institute of Infection, Immunity and Inflammation, College of Medical, Veterinary and Life Sciences, University of Glasgow, Glasgow, G12 8TA, UK.

**Keywords:** E. coli, D-serine, regulation, virulence, type III secretion, sensing, niche adaptation

## Abstract

Sensing environmental stimuli is critically important for bacteria when faced with the multitude of adversities presented within the host. Responding appropriately to these signals and in turn integrating these responses into the regulatory network of the cell allows bacteria to control precisely when and where they should establish colonization. D-serine is an abundant metabolite of the human urinary tract but is a toxic metabolite for *Escherichia coli* that lack a D-serine tolerance locus. Enterohaemorrhagic *E. coli* (EHEC) cannot catabolize D-serine for this reason and colonize the large intestine specifically, an environment low in D-serine. EHEC can however use D-serine sensing to repress colonization thus signaling the presence of an unfavorable environment. In our recent work (Connolly, et al. PLoS Pathogens (2016) 12(1): e1005359), we describe the discovery of a functional and previously uncharacterized D-serine uptake system in *E. coli*. The genes identified are highly conserved in all *E. coli* lineages but are regulated differentially in unique pathogenic backgrounds. The study identified that EHEC, counter-intuitively, increase D-serine uptake in its presence but that this is a tolerated process and is used to increase the transcriptional response to this signal. It was also found that the system has been integrated into the transcriptional network of EHEC-specific virulence genes, demonstrating an important pathotype-specific adaptation of core genome components.

*E. coli* is an extremely diverse gram-negative bacterium that normally forms part of a healthy mammalian gut but occasionally can cause severe disease in humans and animals. Pan-genomic analysis of numerous of *E. coli* isolates has indicated that the organism has an average genome size of around 5000 genes with the core of this being somewhere in the region of 2000 conserved genes. However, the pan-genome size of *E. coli* greatly exceeds this by several fold owing to large scale genomic adaptations to host microenvironments and the horizontal acquisition of foreign DNA, often in the form of plasmids, phages and pathogenicity islands.

EHEC is a major food-borne pathogen of humans causing bloody diarrhea and renal failure in severe cases. The organism is carried asymptomatically in infected ruminant hosts and is indirectly transferred to the human host by contamination of food and water sources. The pathogenicity of this organism is largely due to the carriage of phage-encoded Shiga-like toxins, however, EHEC also encodes a type III secretion system (T3SS) on a large pathogenicity island called the locus of enterocyte effacement (LEE) that is absolutely essential for effective colonization of the host. It has become apparent that numerous *E. coli* sub-types occupy distinct niches in the human intestine and this is largely due to the utilization of nutrient sources and integration of metabolic signals to limit competition in this hostile environment. EHEC, for instance, intimately colonizes the epithelial surface of the large intestine, a site normally devoid of any bacterial life. Intuitively, the mechanisms for this tropism are not dependent on the tissue itself but rather the chemical and metabolic signals present at this particular niche. The mechanisms used by EHEC to sense the correct sites for colonization are not limited to the colon however. Passage through the stomach for instance is a challenging environment and the organism has evolved to integrate regulatory systems such as acid and nitric oxide stress responses into the LEE transcriptional network.

Niche recognition is not restricted to intestinal pathogens however. Certain *E. coli* pathotypes (uropathogenic *E. coli* or UPEC) are capable of ascending the urinary tract after fecal shedding from the host and can go on to cause severe urinary tract infections and septicemia. Surprisingly, UPEC are also extremely competitive in the gut but have evolved to colonize to urinary tract by a combination of attachment mechanisms and the efficient utilization of alternative nutrient sources. One such metabolite is D-serine. D-serine is an abundant amino acid in the urinary tract reaching millimolar concentrations. UPEC utilise the D-serine tolerance locus (*dsdCXA*) to efficiently take up D-serine and catabolize it as a nutrient source. D-serine, however, has also been implicated to act as a regulatory signal capable of altering the transcription of virulence-associated genes in multiple pathogenic bacteria of the urinary tract, such as *E. coli* and the gram-positive pathogen *Staphylococcus saprophyticus*.

D-serine is a toxic metabolite to bacteria that accumulate it intracellularly. Genomic analysis has revealed that the D-serine tolerance locus has been subject to widespread attrition in intestinal *E. coli* and we previously reported that carriage of both an intact *dsdCXA* locus and the LEE pathogenicity island was an extremely rare event. Indeed, D-serine is not found in abundance in the intestinal tract so the need to use it as a carbon source has been lost. Surprisingly, we found that D-serine specifically was capable of transcriptionally repressing expression of the LEE and inhibiting EHEC colonization. This process is concentration dependent and explains why low amounts of D-serine in the gut do not affect colonization using the T3SS. These findings led us to propose that intestinal pathogenic *E. coli* have evolved to specifically lose D-serine tolerance upon acquisition of the LEE, thus restricting the possibility that they may disseminate to the urinary tract. All *E. coli* must pass through the gastrointestinal tract and in theory at the least have the capability of encountering the urinary tract opening but our data suggest that signals beyond the intestine are also extremely important for restricting colonization of unfavorable sites. This also allowed us to suggest one possible reason why UPEC isolates have never acquired the LEE island, in that it would in theory be non-functional in such a D-serine rich environment.

Mechanistically, D-serine repression of the LEE is transcriptional but found to be indirect through modulation of existing regulatory networks. Classically, bacteria respond to environmental signals either by using membrane sensing systems such as two-component sensors or by binding signals directly to transcription factors. In this present study, investigation of core genes that were upregulated under conditions promoting the T3SS identified *yhaO*. This gene encodes a D- and L-serine transporter and is highly conserved in all *E. coli* (Figure 1A). This discovery was surprising given the known toxic effects of D-serine in pathotypes such as EHEC and the loss of the *dsdCXA* locus (encoding its own D-serine transporter) in this background. Despite this, YhaO was functional in both EHEC and UPEC backgrounds as a D-serine transporter. Interestingly, the regulation of *yhaO* in EHEC and UPEC was found to be rather distinct. Using a transcriptional reporter of *yhaO*, exposure to D-serine revealed upregulation in EHEC under conditions promoting the LEE. This process was seemingly counter-intuitive given the toxic nature of D-serine and its effects on transcription of the LEE. Conversely, *yhaO* expression in UPEC was not affected by the presence of D-serine presumably as they already encode an efficient D-serine responsive uptake system (*dsdCXA*). Another surprising finding was that EHEC could actually tolerate high concentrations of D-serine under these conditions. UPEC incurs a growth advantage in the presence of D-serine due to the D-serine tolerance locus. However, a UPEC D-serine deaminase mutant (Δ*dsdA*) could not tolerate D-serine and was static under these conditions. These results demonstrate an EHEC specific adaptation to both growth in the presence of D-serine and the regulation of its uptake (Figure 1B).

**Figure 1:**
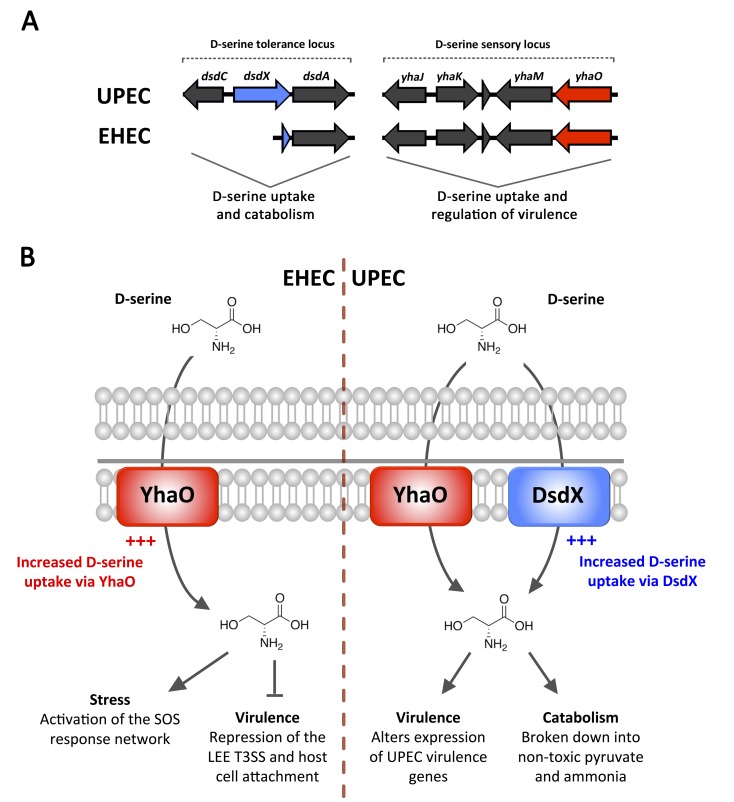
FIGURE 1: The genetics and schematics of D-serine uptake in *E. coli*. **(A) **Genetic organization of the D-serine tolerance and D-serine sensory loci in pathogenic EHEC and UPEC. D-serine tolerance is achieved via uptake (*dsdX* - blue) and catabolism (*dsdA*) of D-serine under the control of a transcriptional regulator, DsdC. This system is truncated in EHEC. D-serine uptake but not catabolism is achieved via YhaO (red), which is also required for virulence of EHEC. **(B)** Model of D-serine uptake and its downstream effects in EHEC and UPEC. UPEC transports D-serine via DsdX and YhaO, but only DsdX is responsive in this genetic background. UPEC uses D-serine as a positive virulence and fitness trait. EHEC regulates D-serine transport via YhaO and accumulates it intracellularly as a signal for stress and regulator of virulence.

The second major finding from this study was a crossover between this core encoded system and regulation of the LEE. *yhaO* lies adjacent to a LysR-type transcriptional regulator (*yhaJ*). We hypothesized that YhaJ may be involved in regulating expression of *yhaO*. Genetic analysis revealed that YhaJ plays a role in regulating *yhaO* directly but not essentially. Additionally, whole transcriptome RNA-seq analysis of both *yhaO* and *yhaJ* mutants in EHEC revealed distinct regulons suggesting that they likely play roles in other processes and not D-serine uptake exclusively. The most striking finding from the RNA-seq analysis, however, was the significant downregulation of the entire LEE island and numerous non-LEE encoded effectors in both the Δ*yhaO* and Δ*yhaJ* backgrounds. In fact, over half of the genes differentially expressed were related to these functions. The mutants were also impaired in their ability to secrete toxic effector proteins and colonize host cells. Furthermore, we demonstrated that YhaJ is capable of directly regulating transcription of the LEE island, specifically by binding upstream of the LEE master regulator and contributing to its activation. These results demonstrate an important pathotype specific adaption of the core genome to perform unique regulatory functions that have a significant impact on the infection process.

The notion that metabolic processes play an unparalleled role in pathogenesis, as much as those related specifically to virulence, has emerged in recent years. It makes perfect sense for a pathogen, or any bacterium, to have systems in place to regulate their behavior in response to their environment. One can certainly consider the burden of “switching on” complex machinery such as the T3SS or similar colonization mechanisms at an inappropriate time and place, both in terms of physical adversity in the environment and also energy requirements. This is particularly clear for EHEC considering the complexity of the T3SS. UPEC, on the other hand, does not harbor one essential virulence factor but rather many mechanisms that contribute to their infection process. UPEC are also very metabolically adaptable, being highly competitive in the gut and later adjusting their transcriptional networks to maximize expression of fitness genes required in the urinary tract. Either scenario represents a key ability of pathogenic *E. coli* - co-operation between core genetic elements and those specifically required for virulence.

Our study has identified a novel and important adaptation of EHEC and further demonstrates the importance of D-serine sensing for this pathogen, however, key questions still remain, such as what is the precise mechanism of D-serine sensing? The discovery of a logical role for YhaO in EHEC challenges assumptions about how bacteria can respond to signals even if they are considered to be detrimental but how is this signal transferred to the genome directly? The precise role of YhaJ is currently under investigation but given its highly conserved nature it likely has a natural role far removed from virulence. Either way, this study is a prime example of how *E. coli* are capable of tailoring their needs and recycling conserved genes for a new and specific purpose. The work highlights how acquisition of virulence factors alone cannot confer pathogenicity without appropriate regulation in response to relevant signals and how this process ultimately contributes to niche recognition within the host.

